# Relationship between EZH2 expression and prognosis of patients with hepatocellular carcinoma using a pathomics predictive model

**DOI:** 10.1016/j.heliyon.2024.e38562

**Published:** 2024-09-28

**Authors:** Xulin Zhou, Muran Man, Min Cui, Xiang Zhou, Yan Hu, Qinghua Liu, Youxing Deng

**Affiliations:** a Department of Oncology, Hefei BOE Hospital, Hefei, PR China; b Department of Oncology, People's Hospital of Shizhong District, Zaozhuang City, Shandong Province, PR China; c Affiliated Hospital Of Jining Medical University (Shanxian Central Hospital), Heze City, Shandong Province, PR China; d People's Hospital of Xinjiang Uygur Autonomous Region Urumqi, Xinjiang, CN, PR China; e Department of Oncology, Deyang People's Hospital, Deyang, Sichuan, CN, PR China

**Keywords:** Hepatocellular carcinoma (HCC), Pathomics, Pathomics score (PS), EZH2, Survival

## Abstract

**Background:**

Enhancer of zeste 2 polycomb repressive complex 2 subunit (EZH2) is overexpressed in hepatocellular carcinoma, promoting tumorigenesis and correlating with poor prognosis. Traditional histopathological examinations are insufficient to accurately predict hepatocellular carcinoma (HCC) survival; however, pathomics models can predict EZH2 expression and HCC prognosis. This study aimed to investigate the relationship between pathomics features and EZH2 expression for predicting overall survival of patients with HCC.

**Methods:**

We analyzed 267 patients with HCC from the Cancer Genome Atlas database, with available pathological images and gene expression data. RNA sequencing data were divided into high and low EZH2 expression groups for prognosis and survival analysis. Pathological image features were screened using mRMR_RFE. A pathological model was constructed using a gradient boosting machine (GBM) algorithm, and efficiency evaluation and survival analysis of the model were performed. The R package “survminer” took the pathomics score (PS) cutoff value of 0.4628 to divide the patients into two groups: high and low PS expression. Survival analyses included Kaplan–Meier curve analysis, univariate and multivariate Cox regression analyses, and interaction tests. Potential pathomechanisms were explored through enrichment, differential, immune cell infiltration abundance, and gene mutation analyses.

**Result:**

EZH2 was highly expressed in tumor samples but poorly expressed in normal tissue samples. Univariate and multivariate Cox regression analyses revealed that EZH2 was an independent risk factor for HCC (hazard ratio [HR], 2.792 and 3.042, respectively). Seven imaging features were selected to construct a pathomics model to predict EZH2. Decision curve analysis showed that the model had high clinical utility. Multivariate Cox regression analysis showed that high PS expression was an independent risk factor for HCC prognosis (HR, 2.446). The Kaplan–Meier curve showed that high PS expression was a risk factor for overall survival.

**Conclusion:**

EZH2 expression can affect the prognosis of patients with liver cancer. Our pathological model could predict EZH2 expression and prognosis of patients with HCC with high accuracy and robustness, making it a new and potentially valuable tool.

## Highlights

• EZH2 overexpression predicts poor survival in liver HCC patients.
• Pathomics model effectively predicts EZH2 levels from H&E-stained images.
• High EZH2 expression linked to increased risk of liver HCC recurrence.
• Machine learning-based pathomics model shows high accuracy in validation.
• Integrated analysis reveals key molecular pathways and immune impacts of EZH2.

## Abbreviations:

HCC: hepatocellular carcinoma
EZH2: Enhancer of zeste 2 polycomb repressive complex 2 subunit
PS: Pathomics score
H&E: Hematoxylin and eosin
TCGA: The Cancer Genome Atlas
GBM: Gradient boosting machine
AUC: Under the curve
ROC: Receiver operating characteristic
DCA: Decision curve analysis
ACC: Accuracy
SPE: Specificity
SEN: Sensitivity
PPV: Positive predictive value
NPV: Negative predictive value
PR: Precision recall
AFP: Alpha fetoprotein

## 1 Introduction

Liver hepatocellular carcinoma (HCC) is among the most common malignancies and the third leading cause of cancer-related death worldwide. By 2025, an estimated 1 million people will develop liver cancer annually [1]. Despite the ongoing advancements in therapeutic technology for cancer worldwide, the 5-year survival rate of HCC remains <20 %, reflecting disease severity and treatment challenges [2]. Hepatitis B virus (HBV) infection is the primary risk factor leading to the development of HCC, accounting for approximately 50 % of these cases. In the West, metabolic syndrome or diabetes-related non-alcoholic steatohepatitis is emerging as a more common risk factor [1]. Surgical resection and liver transplantation are considered the most effective treatments for patients with HCC. However, even after a successful surgical intervention, the risk of recurrence remains significant. Therefore, precise prognostic stratification of patients with HCC, especially during management after surgery, is crucial. To achieve the goals of personalized medicine, identifying and utilizing critical and readily accessible biomarkers to predict disease progression, risk of recurrence, and long-term patient survival has become the focus of current studies [3].

The protein encoded by enhancer of zeste 2 polycomb repressive complex 2 subunit (EZH2) is the catalytic subunit of histone methyltransferase and polycomb inhibitory complex 2. The main function of EZH2 is to catalyze the methylation of H3K27Me3H3 histones, which inhibits the transcription of target genes, such as tumor suppressor genes. Dysregulation of EZH2 is critical to the development and progression of multiple cancer types in mice and humans. Abnormally active or overexpression of EZH2 can lead to changes in gene expression patterns and promote tumor growth and spread and immune evasion through various mechanisms, including suppressing antigen presentation, affecting the migration of immune cells, and enhancing the inhibitory activity of cluster of differentiation (CD) 4 +T regulatory cells. The cumulative effects of these functions make EZH2 an attractive target for tumor therapy [[4], [5], [6]]. Liu et al. showed that mutations and expressional imbalance of EZH2 were associated with melanoma, breast cancer, prostate cancer, lung cancer, liver cancer, psoriasis, and hematological malignancies. The Food and Drug Administration has recently approved tazemetostat, a selective EZH2 inhibitor, for metastatic or advanced epithelioid sarcoma unsuitable for surgical resection [7]. Previous clinicopathological studies have shown that the expression of EZH2 is associated with the progression of HCC and various metastatic characteristics of HCC, including venous invasion, direct liver invasion, and the absence of tumor encapsulation [8].Yang et al. demonstrated that immune-related RNA-binding proteins (RBPs) could predict the prognosis of patients with HCC and constructed a prognostic model. One of the RBP proteins involved in the prediction model is EZH2 [9]. Current methods for detecting EZH2 expression include using fresh tissues, where the gene expression can be detected by qPCR or RNA-seq, and protein levels can be detected by western blotting (WB) and flow cytometry, and using paraffin-embedded tissue specimens, where the expression can be detected by immunohistochemistry or immunofluorescence assays. However, the aforementioned methods are subject to variations due to the operator and antibodies, exhibit inter-laboratory differences, and cannot provide a quantitative and objective assessment [10].

Hematoxylin and eosin (H&E) slide-stained sections are necessary for clinical diagnosis and extracting the most accessible image data. Artificial intelligence is gradually being applied in pathology majors, causing significant changes in pathology [[11], [12], [13]]. Pathological omics (pathomics) refers to the transformation of pathological images into high-fidelity and high-throughput mined data based on artificial intelligence, covering texture, morphology, edge gradient, and biological features. It is used to quantify pathological diagnosis, molecular expression, and disease prognosis [[14], [15], [16]]. Chen et al. constructed a pathological characteristic marker (pathomics signature [gastric cancer]) based on the multiple pathological features of H&E slide-stained sections, namely, pathomics score (PS). PS (GC) could be used as an independent prognostic predictor of gastric cancer [17]. Yang et al. developed a prognostic model based on immune-related genes. The patients were divided into different subgroups according to the genetic model algorithm, and the pathological model was constructed by obtaining the H&E slide staining sections of The Cancer Genome Atlas (TCGA) in the corresponding subgroups. This model can be used to predict the prognosis of patients with HCC [18].

Therefore, this study aimed to verify the relationship between EZH2 expression and prognosis of patients with HCC through survival analysis. Using feature selection and machine learning algorithms, we developed a pathomics model to predict EZH2 expression level in liver HCC tissues. We integrated enrichment and tumor immune microenvironment analyses to explore the possible molecular pathways and biological effects of PS.

## 2 Materials and methods

### 2.1 Data acquisition

**Supplementary Fig. 1:**
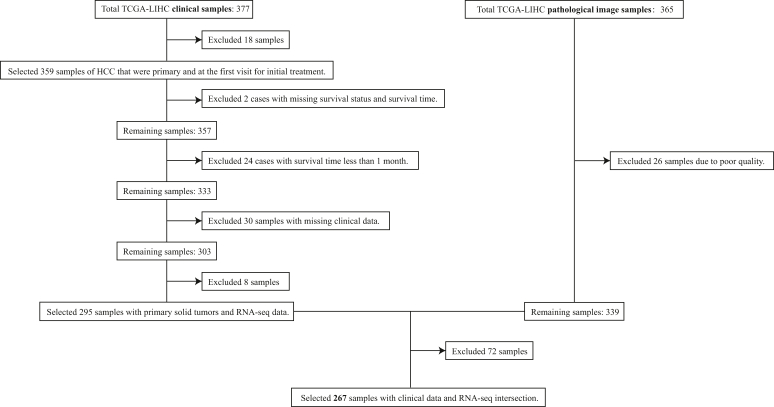
Flow diagram showing inclusion and exclusion processes

The clinical data, sequencing data, and pathological images of patients with liver HCC were downloaded from the TCGA database (https://portal.gdc.cancer.gov/). The screening processes for TCGA-Liver Hepatocellular Carcinoma (LIHC) clinical and pathological data were as follows: (1) Samples were downloaded from TCGA-LIHC clinical data based on the inclusion criterion of primary treatment for liver cancer (n = 359). The exclusion criteria were missing survival status and survival time (n = 2), survival time <1 month (n = 24), samples with missing clinical data (n = 30), non-primary solid tumors without RNA sequencing (RNA-seq) (n = 8), and having TCGA-LIHC clinical data (n = 295). 2. A total of 365 samples were downloaded from the TCGA-LIHC pathological image database, 26 substandard samples were excluded, and 339 patients with TCGA-LIHC pathological image data were obtained. Finally, TCGA-LIHC clinical data and TCGA-LIHC pathological image data were subjected to intersection analysis, and 267 patients with clinical data and pathological images were included in this study. The flow diagram for inclusion and exclusion processes is presented in Supplementary Figure 1.

We included the following variables as covariates: age (<60 years vs. ≥ 60 years), sex (female vs. male), pathological stage (I/II vs. III/IV), hepatic inflammation (none vs. unknown vs. mild/severe), histological grade (G1/G2 vs. G3/G4), ablation–embolization (no vs. unknown vs. yes), vascular invasion (none vs. unknown vs. micro/macro), alpha-fetoprotein level (<400 vs. unknown vs. ≥ 400), residual tumor (R0 vs. R1/R2/RX), and pharmaceutical therapy (no vs. yes).

All participants in TCGA provided written informed consent, along with necessary ethics approval in the original study.

### 2.2 Analysis of clinicopathological features and prognosis based on EZH2 expression

The RNA-seq data in level 3 HTSeqFPKM format in the TCGA (https://portal.gdc.cancer.gov/) LIHC (liver cancer) project were used. The RNA-seq data in fragments per kilobase per million format were transformed into log2, and the difference between tumors and normal tissues was obtained. The cutoff for the expression level of EZH2 was taken as 1.61 using the R package “survminer” to divide the patients into groups with high or low EZH2 expression. Survival was calculated using Kaplan–Meier curves, and risk factors affecting patient outcomes were analyzed using Cox regression.

### 2.3 Pathomics feature extraction and model establishment

#### 2.3.1 Intersection of TCGA pathology samples

There were 267 samples with available pathological images, gene matrices, and complete clinical data. The data were randomly divided into a training set and a validation set in a 7:3 ratio, and the group differences between the training and validation sets were analyzed. The between-group differences in clinical variables among the datasets were analyzed. There were 187 cases in the training set and 80 cases in the validation set. The *P* value of the intergroup difference analysis was >0.05, indicating that the training set was close to the baseline condition of patients in the validation set and was comparable between groups. Baseline data are provided in Supplementary Table 1.

#### 2.3.2 Image segmentation and feature extraction

Pathological image acquisition: Pathological images were downloaded from the TCGA (https://tcga-data.nci.nih.gov/tcga/) database and processed using formalin and paraffin-embedded pathological tissue sections in svs format with a maximum magnification of 20× or 40 × (H&E-stained histopathological images [20 × or 40× magnification]) [19,20].

Pathological image processing and segmentation: The tissue regions of the pathological sections were obtained using the OTSU algorithm (https://opencv.org/). The OTSU algorithm, also known as the maximum inter-class variance method, is a threshold algorithm for image binary segmentation that uses a threshold to divide the image into two parts: the unwanted background and the tissue region required for research [21]. The 40 × image was divided into a plurality of 1024 × 1024 pixel sub-images (sub-images), the 20 × image was divided into a plurality of 512 × 512 pixel sub-images, and the upsampling was 1024 × 1024 pixels. The pathologist then reviewed it to exclude sub-images with poor image quality (contamination, blurred image, and >50 % blank area). Ten sub-images were randomly selected from each pathology image for subsequent analysis [19,20].

**Fig. 1:**
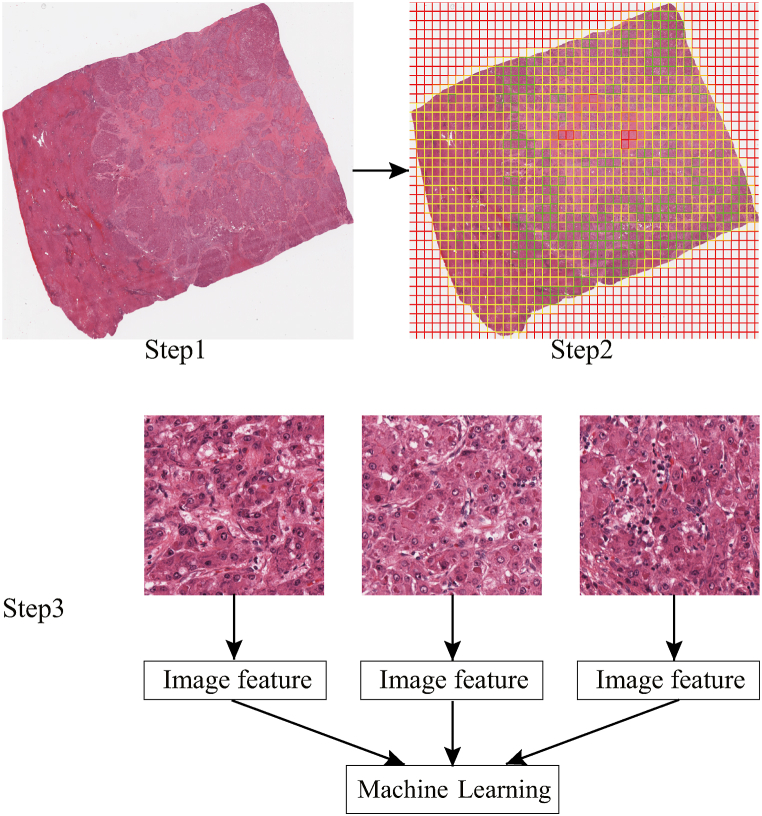
Schematic diagram of the pathological image feature extraction process. Step 1, raw image collection; Step 2, image segmentation; and Step 3, feature extraction.

Feature extraction: Using the PyRadiomics (https://pyradiomics.readthedocs.io/en/latest/) open-source package, the sub-image was standardized, and 93 original features (including first- and second-order features) were extracted. In total, 465 features were obtained by extracting high-order feature wavelet. After extracting features from the 10 sub-images of each patient, the corresponding mean (the average value) was taken as the pathomics feature for each sample for subsequent data analysis [14,22,23]. The histopathological eigenvalues of the training set (465 features extracted using the pyradiomics package) were standardized using z-scores, and the mean and standard deviation of the training set were used to standardize the validation set. A schematic of the image feature extraction process is shown in Fig. 1.

#### 2.3.3 Construction of the pathomics model

Using the “mRMRe” package of R, the top 20 features were selected using the maximum relevance, minimum redundancy (mRMR) method, and the best feature subset was further selected by recursive feature elimination (RFE). Finally, the selected pathomics features were modeled using the gradient boosting machine (GBM) algorithm.

### 2.4 Model evaluation

We evaluated model efficacy using accuracy (ACC), specificity (SPE), sensitivity (SEN), positive predictive value (PPV), and negative predictive value (NPV). Precision recall (PR) curves were used to comprehensively assess the performance, PR-area under the curve (AUC) was defined as the average of the ACC calculated for each coverage threshold, and the PR curve was better in the upper right corner. The receiver operating characteristic (ROC) curve was used to evaluate the overall performance of the pathomics model; the larger the area under the ROC-AUC curve, the higher the upper left corner of the curve, and the better the model effect. Calibration of the pathomics prediction model was evaluated by plotting the calibration curve and performing the Hosmer–Lemeshow goodness-of-fit test. Quantifying the comprehensive performance of the pathomics prediction model using the Brier score, the smaller the value, the better the consistency of the model predictions. The final decision curve analysis (DCA) revealed the clinical benefits of the pathomics prediction model. Group differences in PS and EZH2 expression were analyzed using the Wilcoxon test, and the results were visualized using the R package Ggpubr.

### 2.5 Pathological omics mechanism analysis

The pathway enrichment scores of the Kyoto Encyclopedia of Genes and Genomes (KEGG) pathway gene sets and hallmark gene sets in each sample were calculated using gene set variation analysis (GSVA) for the expression matrix of the 267 patients with HCC in the TCGA. Differential analysis of the PS height grouping was performed using the R package “limma,” and the top 30 pathways were visualized with |t | = 1 as the cutoff value. The variability in immune-related gene expression between the PS groups was analyzed using the Wilcoxon test. The gene expression matrix of the liver cancer samples was uploaded to the CIBERSORTx database (https://cibersortx.stanford.edu/), and the immune cell infiltration of each sample was calculated. Mutation data from TCGA-LIHC patients were downloaded from the TCGA data portal, data for somatic variants were stored in mutation annotation format, and the mutation data were analyzed using the R package maftools.

### 2.6 Statistical analyses

Statistical analyses were performed using R software (version 4.1.0). Kaplan–Meier survival curves were drawn using the “survival” package of R language to show the change in survival rates in different groups for each variable, and the significance test of survival between each group was performed using the log-rank test. The risk factors affecting overall survival (OS) were identified using univariate and multivariate Cox regression analyses. Exploratory subgroup analysis was performed using univariate Cox regression, and interaction analysis was performed using the likelihood ratio test. A *P* value < 0.05 was considered statistically significant.

## 3 Results

### 3.1 Analysis of clinical characteristics between the EZH2 groups

**Fig. 2:**
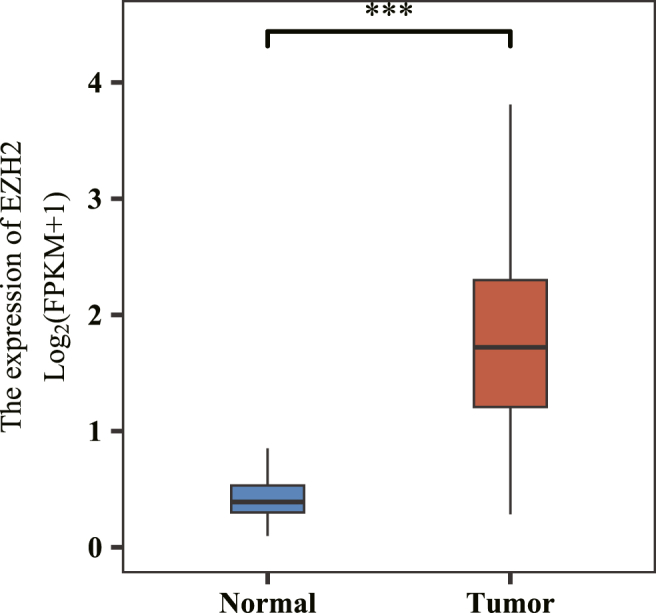
Enhancer of zeste 2 polycomb repressive complex 2 subunit (EZH2) analysis of group differences between tumors and normal tissues (identification of significance: ns, *P =* 0.05; ∗*P* < 0.05; ∗∗*P* < 0.01, ∗∗∗*P* < 0.001).

In total, 267 patients from the TCGA project were included, and the cutoff expression level was 1.61, dichotomizing patients into the high (n = 140) and low (n = 127) EZH2 groups. Analysis of group differences between EZH2 tumors and normal tissues suggested that EZH2 expression was higher in the tumor group than in the normal group, and the median difference between the two groups was 1.303 (1.129–1.496, *P* < 0.001) (Fig. 2). No statistically significant differences were found in the clinical factors between the two groups, except for the covariate histological grade (*P* = 0.002, Table 1).

**Table 1:** EZH2 baseline data between the high and low expression groups.

Variables	Total (n = 267)	Low (n = 127)	High (n = 140)	*P*
Pathological stage, n (%)				0.088
Stage I/II	203 (76)	103 (81)	100 (71)	
Stage III/IV	64 (24)	24 (19)	40 (29)	
Histological grade, n (%)				0.002
G1/G2	164 (61)	91 (72)	73 (52)	
G3/G4	103 (39)	36 (28)	67 (48)	
Pharmaceutical therapy, n (%)				0.341
No	240 (90)	117 (92)	123 (88)	
Yes	27 (10)	10 (8)	17 (12)	
Ablation–embolization, n (%)				0.872
No	203 (76)	95 (75)	108 (77)	
Unknown	45 (17)	22 (17)	23 (16)	
Yes	19 (7)	10 (8)	9 (6)	
Sex, n (%)				0.589
Female	81 (30)	36 (28)	45 (32)	
Male	186 (70)	91 (72)	95 (68)	
AFP, n (%)				0.597
∼399	138 (52)	65 (51)	73 (52)	
400∼	69 (26)	36 (28)	33 (24)	
Unknown	60 (22)	26 (20)	34 (24)	
Age, n (%)				0.233
∼59	129 (48)	56 (44)	73 (52)	
60∼	138 (52)	71 (56)	67 (48)	
Hepatic inflammation, n (%)				<0.001
Mild/severe	86 (32)	38 (30)	48 (34)	
None	93 (35)	58 (46)	35 (25)	
Unknown	88 (33)	31 (24)	57 (41)	
Vascular invasion, n (%)				0.046
Micro/macro	76 (28)	38 (30)	38 (27)	
None	153 (57)	78 (61)	75 (54)	
Unknown	38 (14)	11 (9)	27 (19)	
Residual tumor, n (%)				0.257
R0	246 (92)	120 (94)	126 (90)	
R1/R2/RX	21 (8)	7 (6)	14 (10)	

Abbreviations: EZH2, enhancer of zeste 2 polycomb repressive complex 2 subunit; AFP, alpha fetoprotein.

### 3.2 Associations between overall survival and clinicopathological characteristics using Cox regression

**Fig. 3:**
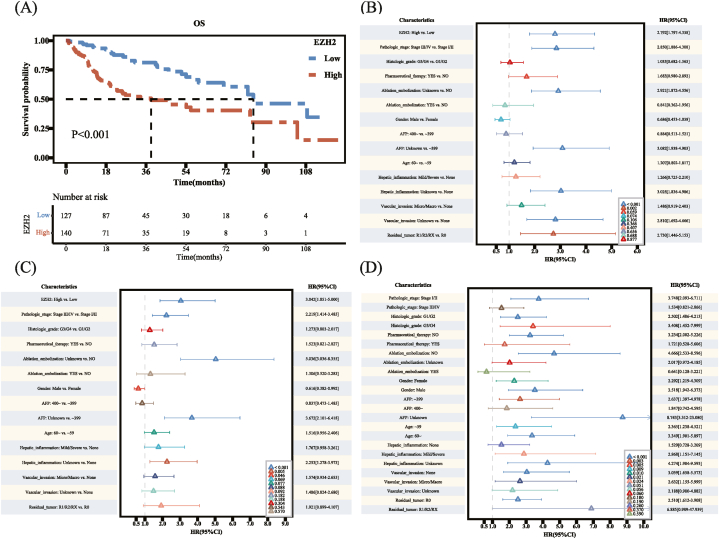
Enhancer of zeste 2 polycomb repressive complex 2 subunit (EZH2) analysis of clinical characteristics between the high and low groups. (A) Kaplan–Meier survival curve showing the change in survival rate of different groups. (B) Results of univariate Cox analysis. (C) Results of multivariate Cox analysis. (D) Subgroup analysis and interaction test.

The Kaplan–Meier survival curve showed that the median survival times were 84.4 months and 38.3 months in the EZH2 low and high expression groups, respectively. High EZH2 expression was associated with OS deterioration (*P* < 0.001, Fig. 3A). In the univariate analysis, high EZH2 expression was a risk factor for OS (hazard ratio [HR] = 2.792; 95 % confidence interval [CI], 1.797–4.338; *P* < 0.001). In the multivariate analysis, high EZH2 expression (HR = 3.042; 95 % CI, 1.851–5.000; *P* < 0.001) was a statistically significant risk factor for OS (Fig. 3B and C).

### 3.3 Subgroup analysis and interaction testing

In the subgroup analysis, in the subgroup aged <60 years, increased EZH2 expression was a risk factor for OS (HR = 2.365; 95 % CI, 1.238–4.521; *P* = 0.009 [statistically significant]). In the subgroup aged >60 years, increased EZH2 expression was also a risk factor for OS (HR = 3.349; 95 % CI, 1.901–5.897; *P* < 0.001 [statistically significant]). The *P* test for interaction was >0.05. There was no significant interaction between EZH2 and the different age subgroups. In the same subgroup, increased EZH2 expression was a risk factor for OS, regardless of sex (Fig. 3D).

### 3.4 Pathomics feature extraction and model establishment

**Fig. 4:**
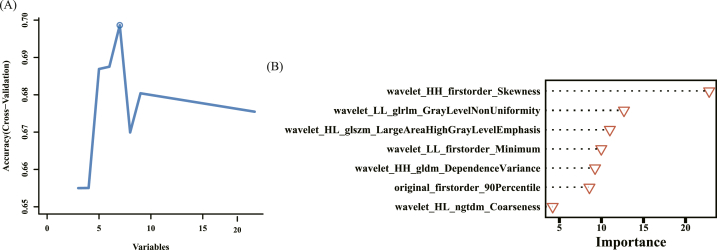
Feature screening and gradient boosting machine (GBM) model establishment. (A) Schematic diagram of feature screening. (B) The importance of seven pathomics features in the GMB algorithm.

Seven pathomics features were finally obtained by mRMR-RFE feature screening: wavelet _ HH _ firstorder _ Skewness, wavelet_LL_glrlm_GrayLeveNonUniformity, wavelet_HL_glazm_LargeAreaHighGrayLevelEmphasis, wavelet_LL_firstorder_Minimum, wavelet_HH_gldm_DependenceVariance, original_firstorder_90Percentile, and wavelet_HL_ngtdm_Coarseness (Fig. 4A). A pathomics model was constructed using the GBM algorithm, and its efficacy was evaluated. Fig. 4B shows the importance of the selected features in the GBM algorithm.

### 3.5 Model evaluation and intergroup difference analysis

#### 3.5.1 Efficiency evaluation

**Fig. 5:**
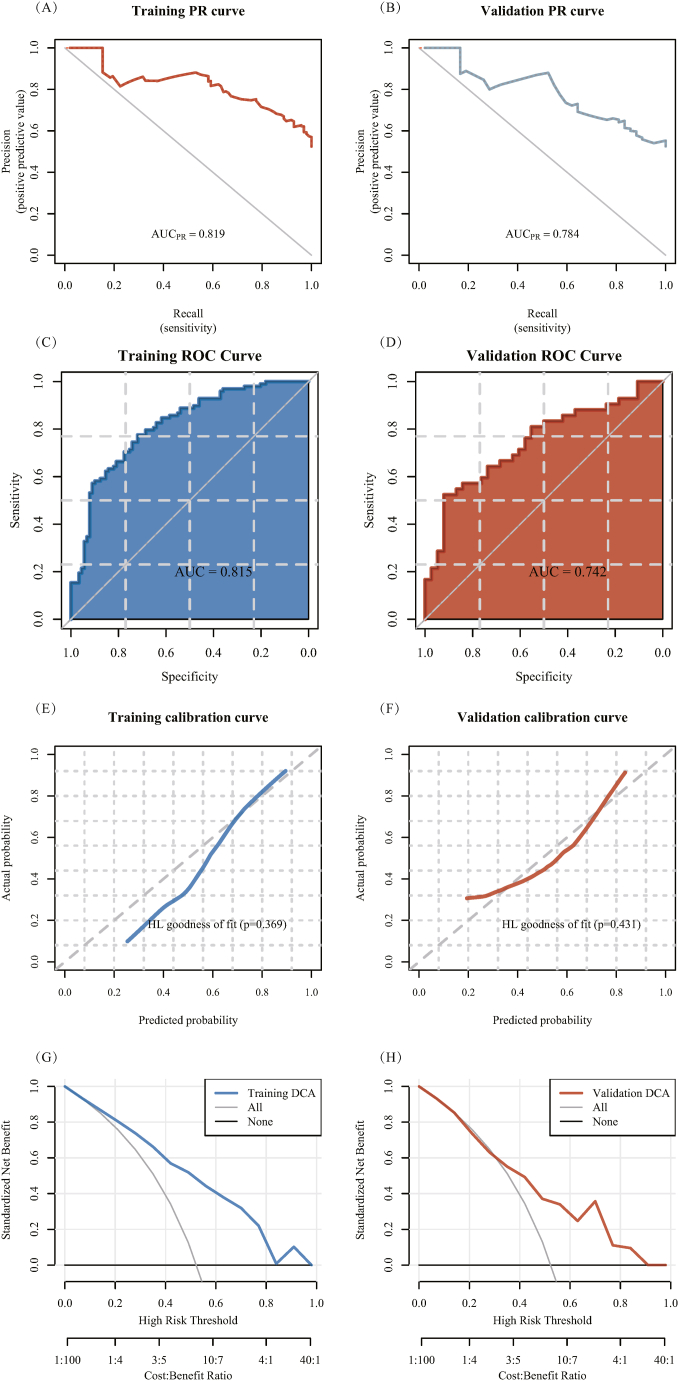
Evaluation of model efficacy. (A–B) Precision-recall (PR) curve for the training and validation sets: The X-axis is the coverage (recall), namely, the true positive rate, and the Y-axis is the accuracy (precision). (C–D) The receiver operating characteristic (ROC) curves of the training and validation sets: The X-axis of the ROC curve is the false positive rate (1-specificity), and the Y-axis is the true positive rate (sensitivity). (E–F) Calibration curves and Hosmer–Lemeshow goodness-of-fit tests for the training and validation sets. (G–H) Decision curve analysis of the training and validation sets; The Y-axis measures the net gain, and the decision curve analysis represents the pathomics model.

The pathomics model had a good predictive effect. The ACC, SPE, SEN, PPV, and NPV in the training and validation sets were 0.749, 0.719, 0.776, 0.752, and 0.744 and 0.713, 0.921, 0.524, 0.88, and 0.636, respectively. As shown in the precision-recall (PR) curve (Fig. 5A and B), the PR-AUCs of the training and validation sets were 0.819 and 0.784, respectively. The subject ROC curve of the training and validation sets were 0.815 and 0.742, respectively (Fig. 5C and D). This indicates that the comprehensive evaluation of the model performance was good. The Brier scores for the training and validation sets were 0.179 and 0.205, respectively (Brier score <0.25). The calibration curve and Hosmer–Lemeshow goodness-of-fit test showed that there was a good agreement with the true values of the histopathological prediction model for high gene expression (Fig. 5E and F). The *P* values of the training and validation sets were 0.369 and 0.431, respectively (*P* > 0.05). The DCA showed that the model had high clinical practicability (Fig. 5G and H).

#### 3.5.2 Differences in the pathomics score (PS) distribution of the gradient boosting machine model between the high and low EZH2 expression groups

**Fig. 6:**
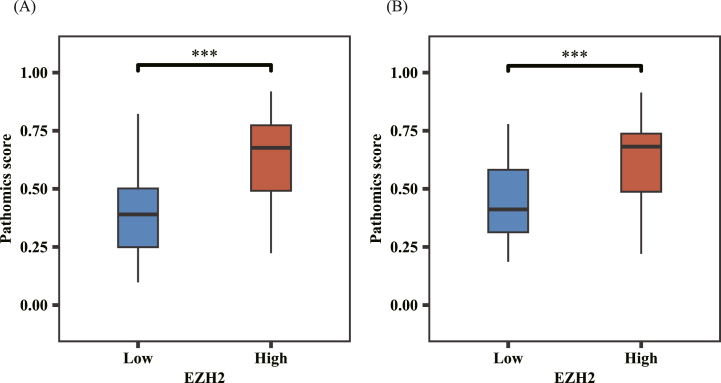
Distribution of pathomics score values in the training (Fig. 6A) and validation sets (Fig. 6B) (identification of significance: ns, *P* = 0.05; ∗*P* < 0.05; ∗∗*P* < 0.01; ∗∗∗*P* < 0.001).

Differences between PSs in the training and validation sets were compared using the Wilcoxon test. The pathomics model output the probability PS to predict gene expression levels. In the training set (Fig. 6A), PS distribution was significantly different between the high and low EZH2 expression groups (*P* < 0.001). The EZH2 high expression group had higher PS than the EZH2 low expression group. In the validation set (Fig. 6B), PS distribution was significantly different between the high and low EZH2 expression groups, and the PS value of the high EZH2 expression group was significantly higher than that of the low EZH2 expression group (*P* < 0.001).

### 3.6 Clinical characteristics of high and low histopathological scores

Taking the cutoff value of the predicted value of the PS of the GBM model as 0.4628, the patients were divided into a high expression group (n = 155) and a low expression group (n = 112). The histopathological scores and clinical data were combined to analyze the clinical features of the PS groups. There was no significant difference in the distribution of histological grade or sex between the high and low PS groups (*P* < 0.01, Table 2).

**Table 2:** PS baseline data between the high and low expression groups.

Variables	Total (n = 267)	Low (n = 112)	High (n = 155)	*P*
Pathological stage, n (%)				0.065
Stage I/II	203 (76)	92 (82)	111 (72)	
Stage III/IV	64 (24)	20 (18)	44 (28)	
Histological grade, n (%)				1
G1/G2	164 (61)	69 (62)	95 (61)	
G3/G4	103 (39)	43 (38)	60 (39)	
Pharmaceutical therapy, n (%)				0.453
No	240 (90)	103 (92)	137 (88)	
Yes	27 (10)	9 (8)	18 (12)	
Ablation–embolization, n (%)				0.656
No	203 (76)	82 (73)	121 (78)	
Unknown	45 (17)	21 (19)	24 (15)	
Yes	19 (7)	9 (8)	10 (6)	
Sex, n (%)				0.888
Female	81 (30)	35 (31)	46 (30)	
Male	186 (70)	77 (69)	109 (70)	
AFP, n (%)				0.006
∼399	138 (52)	68 (61)	70 (45)	
400∼	69 (26)	29 (26)	40 (26)	
Unknown	60 (22)	15 (13)	45 (29)	
Age, n (%)				0.033
∼59	129 (48)	45 (40)	84 (54)	
60∼	138 (52)	67 (60)	71 (46)	
Hepatic inflammation, n (%)				<0.001
None	93 (35)	52 (46)	41 (26)	
Mild/severe	86 (32)	36 (32)	50 (32)	
Unknown	88 (33)	24 (21)	64 (41)	
Vascular invasion, n (%)				<0.001
None	153 (57)	77 (69)	76 (49)	
Micro/macro	76 (28)	30 (27)	46 (30)	
Unknown	38 (14)	5 (4)	33 (21)	
Residual tumor, n (%)				0.127
R0	246 (92)	107 (96)	139 (90)	
R1/R2/RX	21 (8)	5 (4)	16 (10)	

Abbreviations: PS, pathomics score; AFP, alpha fetoprotein.

### 3.7 Associations between overall survival and clinicopathological characteristics using Cox regression

**Fig. 7:**
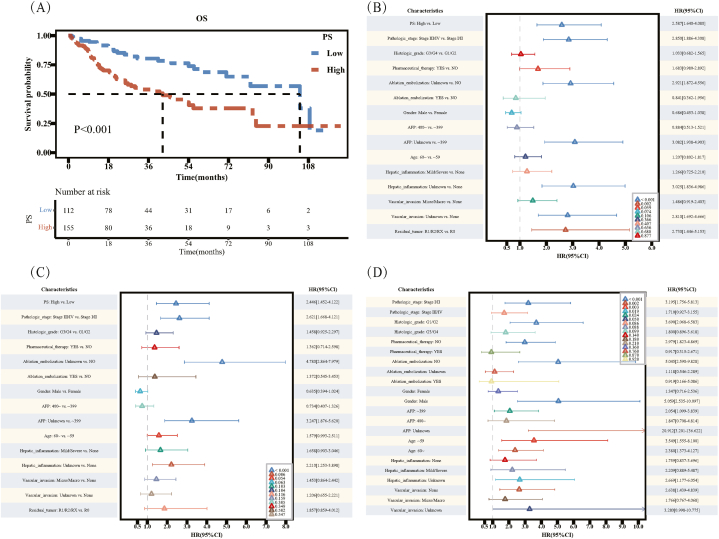
Pathomics score (PS) analysis of clinical characteristics between the high and low PS groups. (A) Kaplan–Meier survival curve showing the change in survival rate of different groups. (B) Results of univariate Cox analysis. (C) Results of multivariate Cox analysis. (D) Subgroup analysis and interaction test.

The Kaplan–Meier curve showed that the median survival times were 42.37 and 104.17 months in the high and low PS groups, respectively. A high PS was significantly associated with a worse OS (*P* < 0.001, Fig. 7A). High PS expression was a risk factor for OS in the univariate analysis (HR = 2.587; 95 % CI, 1.64–4.08; *P* < 0.001 [statistically significant]) (Fig. 7B). In the multivariate analysis, high PS expression (HR = 2.446; 95 % CI, 1.452–4.122; *P* < 0.001) was a statistically significant risk factor for OS (Fig. 7C).

### 3.8 Subgroup analysis and the interaction experiments

In the subgroup analysis, in the subgroup aged <60 years, elevated PS was a risk factor for OS (HR = 3.549; 95 % CI, 1.555–8.1; *P* = 0.003 [statistically significant]). In the subgroup aged >60 years, elevated PS was also a risk factor for OS (HR = 2.38; 95 % CI, 1.373–4.127; *P* = 0.002 [statistically significant]). The *P*-value of the interaction test was 0.4. There was no significant interaction between PS and different age subgroups. In other words, the effect of PS on OS was similar between the two age subgroups (Fig. 7D).

### 3.9 Pathomics model mechanism

Model prediction results between the PS groups: GSVA enrichment analysis, differential analysis of immune-related genes, differential analysis of PS group immune cell abundance, and gene mutation analysis had the following results.

#### 3.9.1 Model prediction results of gene set variation enrichment analysis between the high and low PS groups

**Fig. 8:**
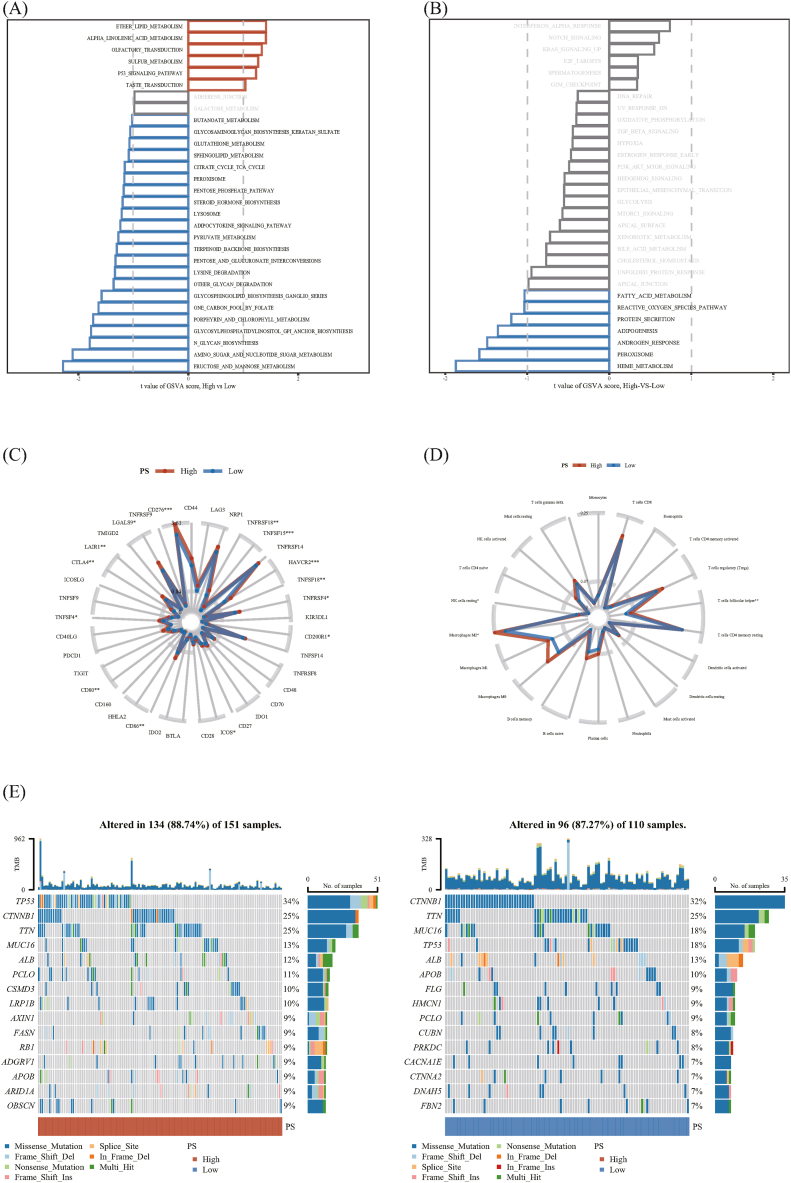
Pathomics mechanism analysis. (A) Kyoto Encyclopedia of Genes and Genomes pathway gene set enrichment analysis. (B) Hallmark gene set enrichment analysis. (C) Differential analysis of immune-related genes. (D) Differential analysis of immune cell abundance. (E) Gene mutation difference analysis (identification of significance: ns, *P* ≥ 0.05; ∗*P* < 0.05; ∗∗*P* < 0.01; ∗∗∗*P* < 0.001).

To clarify the mechanism underlying the pathomics, we performed GSVA and classified the PS of the GBM pathomics model as low/high dichotomous variables (PS). KEGG pathway gene set analysis revealed 186 pathways and 50 pathways in hallmark gene set enrichment analysis. The results showed that in the KEGG gene set, the high PS group was significantly enriched in signaling pathways, such as P53 signaling_pathway, sulfur metabolism, olfactory transduction, alpha linolenic acid metabolism, and ether lipid metabolism (Fig. 8A). In the hallmark gene set, the low PS group was significantly enriched in signaling pathways, such as peroxisome, heme metabolism, androgen response, and adipogenesis (Fig. 8B).

#### 3.9.2 Differential analysis of PS and immune-related genes

Differential analysis of immune-related genes can assist in evaluating the target molecules of the pathomics mechanism of HCC. The Wilcoxon test was used to analyze the differences in the expression of 37 immune-related genes between the high and low PS groups [24]. *P* < 0.05 was considered statistically significant. The gene expression levels of CD276, tumor necrosis factor superfamily member 15 (TNFSF15), and hepatitis A virus cellular receptor 2 (HAVCR2) significantly increased in the high PS expression group (*P* < 0.001, Fig. 8C).

#### 3.9.3 Differential analysis of immune cell abundance of the high and low PS groups

Differences in the degree of immune cell infiltration between the high and low PS expression groups were analyzed using the Wilcoxon rank-sum test. The results showed that the infiltration of follicular helper T cells and macrophages M2 was higher in the high PS group than in the low PS group (*P* < 0.05, Fig. 8D).

#### 3.9.4 Model prediction results of PS gene mutation analysis between the high and low PS groups

The intersection sample size of the mutation and pathology omics data in TCGA-LIHC patients was 261. Fig. 8E presents the top 15 mutated genes with the highest frequency of mutations. The highest to lowest mutation types were missense mutation (Missense_Mutation), frameshift deletion mutation (Frame _ Shift _ Del), and nonsense mutation (Nonsense_Mutation). Both TTN and CTNNB1 had >20 % mutation rates in the high and low PS groups. TP53 and PCLO had higher mutation rates in the high PS group than in the low PS group.

## 4 Discussion

Based on a comprehensive pathomics-molecular analysis of pathological images, we investigated the correlation between pathomics features and EZH2 expression levels in the diagnosis, treatment, and OS of HCC. Our results demonstrated that histopathological imaging features were associated with differences in EZH2 expression levels and OS in liver cancer.

The prognosis of HCC is poor, with an insidious onset, and HCC is usually diagnosed first at a late stage. Generally, only 5%–15 % of patients with early-stage HCC are suitable for surgical resection. Compared with left liver resection, right liver resection is associated with a higher risk of postoperative complications; thus, accurate prognosis is the key to HCC treatment [25]. EZH2 is closely associated with poor prognosis and the promotion of HCC progression. It is overexpressed in HCC tissues. It is extensively involved in the proliferation and metastasis of HCC cells, inhibits immune cell function, and participates in the immune evasion of HCC cells [26]. This suggests that EZH2 may be a risk factor associated with the prognosis of liver cancer [27,28]. The present study demonstrated that a short median survival time in patients with high EZH2 expression was associated with a worse prognosis of HCC. We believe that the expression levels of EZH2 based on pathomics will facilitate nuanced clinical judgment.

In recent years, advancements in molecular biology, multi-omics research, and artificial intelligence technologies have benefited cancer researchers [29,30]. H&E stained slides are widely used due to their low cost and universal applicability, and the combination of feature extraction from H&E staining images with artificial intelligence has propelled the development of pathomics [12]. In particular, the successful application of pathomics to tumors, including automated diagnosis, risk assessment, and survival prediction, has shown several interesting results in terms of patient outcomes [17,31,32]. Kim et al. developed a model that combined clinical information, deep learning, and pathomics, and its predictive performance for BRAF mutations achieved an ROC-AUC value of 0.71 [33]. Pathological studies can be used to establish a predictive model for total survival in HCC. Yang et al. used other pathomics techniques to mine pathological markers of patients with liver HCC, identify six immune-related genes affecting prognosis, and construct a prognostic risk prediction model. In the training and validation sets, the ROC-AUC values of this model were 0.709 and 0.852, respectively, indicating a good predictive value [18]. In this study, the TCGA pathological intersection samples of patients with liver cancer were extracted, seven features were screened using the mRMR _ RFE algorithm, and a prediction model was constructed using the GBM algorithm. The AUC values of the ROC curves of the training and validation sets were 0.815 and 0.742, respectively. These results suggest that the model predicting EZH2 expression level performs well. Targeted drugs have been developed for EZH2 in liver cancer therapy [7,34,35], such as DZnep and GSK126, which have been validated HCC cell lines and xenograft models [36,37]. Xiao G et al. suggested that EZH2 negatively regulates PD-L1 expression in HCC and may serve as a potential therapeutic target for combination immunotherapy in immune-activated HCC [38]. The objective, quantitative, and accurate prediction of EZH2 through pathomics can provide a basis for screening populations with potential benefits for immunotherapy in the future. PS calculated using the pathological model indicated that a high PS was significantly associated with OS deterioration (*P* < 0.001), and Cox regression in the univariate and multivariate analyses suggested that a high PS was an independent risk factor for OS. The effect of the PS level was similar between the different age subgroups.

We analyzed the pathomechanism underlying high and low PS in model prediction results. Differential analysis of immune-related genes suggested that the expression of genes such as CD276, TNFSF15, and HAVCR2 was significantly higher in the high PS group. CD276 promotes epithelial-mesenchymal transition (EMT) and HCC invasion through the JAK2/STAT3/slug pathway [39] and induces M2 polarization of tumor-associated macrophages (TAMs) to promote an immunosuppressive tumor microenvironment (TME) in a STAT3-dependent manner [40]. CD276 expression correlates with aggressive phenotypes such as vascular invasion, advanced tumor staging, and the metastatic potential of HCC cell lines [41]. TNFSF15, or vascular endothelial growth inhibitor (VEGI or TL1A), is involved in regulating vascular homeostasis [42]. Interestingly, it plays a role in tumor suppression, promotes the differentiation and polarization of macrophages towards the M1 phenotype, and inhibits tumor growth [43]. Al-Danakh et al. indicated TNFSF15 as a tumor suppressor gene associated with disparities in age-related survival and its link to pathological staging and various immune statuses [44]. HAVCR2 or TIM-3 [45], plays a key role in immune regulation and is an independent indicator of poor prognosis in liver cancer [46]. The expression of HAVCR2 is significantly increased on infiltrating tumor tissues of CD4^+^ and CD8^+^ T cells, which may play an important role in progression, invasion, and metastasis [47,48]. These molecules have been mentioned in studies on cancer immunotherapy and prognostic evaluation [[49], [50], [51]]. Differential analysis of the abundance of immune cell infiltration suggested that follicular helper T (Tfh) cells and macrophages M2 were higher in the high PS group than in the low PS group. The study by Gutierrez-Melo and Baumjohann are different from the present study, suggesting that an increased frequency of Tfh cells is usually associated with unfavorable outcomes and that an increased frequency of Tfh cells in non-lymphocyte-derived solid organ tumors is generally associated with better prognosis [52]. Mehla et al. showed that some macrophage metabolic pathways in the TME were transformed into inflammatory (M1) or regulatory (M2) subtypes, whereas M2-like cells promoted tumor growth by inducing immunosuppression [53,54]. Gene mutation analysis suggested that the mutation rates of both TTN and CTNNB1 were >20 % in the high and low PS groups. These two molecules have been studied as potential diagnostic and prognostic biomarkers for several cancers [55]. Hu et al. suggested that the CTNNB1 mutation could regulate the metabolic phenotype to affect HCC prognosis [56]. The mutation rate of TP53 and PCLO genes was higher in the high PS group than in the low PS group, which may be related to these two molecules being risk factors for tumor development. Long et al. demonstrated that TP53-related liver cancer could be used to establish an immune prognostic model [57]. However, Moul et al. suggested that the most common somatic mutations associated with lipid metabolism in liver cancer were CTNNB1, TTN, TP53, ALB, MUC16, and PCLO [58]. This coincides with our research findings.

Our study has some limitations. First, all images were derived from the public TCGA dataset, which inevitably has differences in image quality that may affect the prediction analysis. Second, this was a retrospective study with a relatively small sample size; therefore, its generalizability remains to be investigated. Additionally, image reconstruction algorithms, preprocessing methods, individual differences, and feature extraction algorithms can affect the stability and reproducibility of pathomics features.

## 5 Conclusions

Our findings indicate that EZH2 expression is an independent prognostic factor for HCC. Pathomics models based on HE staining can more accurately predict the expression of EZH2 and the prognosis of patients with HCC and hold potential application value for the precision treatment of HCC.

## Data availability statement

The data used for the study are available online at: https://portal.gdc.cancer.gov/.

## Ethical approval statement

All participants in TCGA provided written informed consent, along with necessary ethics approval in the original study.

## Conflict of interest disclosure

The authors have no conflicts of interest to declare.

## Funding

This study was supported by the Sichuan Medical (Youth Innovation) Research Project “Insight of Allitridum into Molecular Mechanism of Apoptosis Induction Mediated by hnRNP K via Regulating Bcl-2 Gene Expression in Hepatic Stellate Cells” (project number: S22051,2023.01–2025.12) in 2022.

## CRediT authorship contribution statement

**Xulin Zhou:** Writing – original draft, Data curation. **Muran Man:** Data curation. **Min Cui:** Formal analysis. **Xiang Zhou:** Visualization. **Yan Hu:** Visualization. **Qinghua Liu:** Supervision, Funding acquisition. **Youxing Deng:** Supervision.

## Declaration of competing interest

The authors declare that they have no known competing financial interests or personal relationships that could have appeared to influence the work reported in this paper.
