# Supplementary material for: Relationship between EZH2 expression and prognosis of patients with hepatocellular carcinoma using a pathomics predictive model
Source: Heliyon. 2024 Sep 28;10(20):e38562. doi: 10.1016/j.heliyon.2024.e38562 (PMC11619983; doi:10.1016/j.heliyon.2024.e38562)
Supplement: Multimedia component 1 [file mmc1.docx]

Supplementary Table 1 Baseline patient data for the training and verification sets

| Variables | Total (n = 267) | Train (n = 187) | Validation (n = 80) | ^*^*P* |
| --- | --- | --- | --- | --- |
| EZH2, n (%) |  |  |  | 1 |
| Low | 127 (48) | 89 (48) | 38 (48) |  |
| High | 140 (52) | 98 (52) | 42 (52) |  |
| Pathological stage, n (%) |  |  |  | 0.176 |
| Stage I/II | 203 (76) | 147 (79) | 56 (70) |  |
| Stage III/IV | 64 (24) | 40 (21) | 24 (30) |  |
| Histological grade, n (%) |  |  |  | 0.861 |
| G1/G2 | 164 (61) | 116 (62) | 48 (60) |  |
| G3/G4 | 103 (39) | 71 (38) | 32 (40) |  |
| Pharmaceutical therapy, n (%) |  |  |  | 1 |
| No | 240 (90) | 168 (90) | 72 (90) |  |
| Yes | 27 (10) | 19 (10) | 8 (10) |  |
| Ablation–embolization, n (%) |  |  |  | 0.073 |
| No | 203 (76) | 147 (79) | 56 (70) |  |
| Unknown | 45 (17) | 31 (17) | 14 (18) |  |
| Yes | 19 (7) | 9 (5) | 10 (12) |  |
| Sex, n (%) |  |  |  | 0.721 |
| Female | 81 (30) | 55 (29) | 26 (32) |  |
| Male | 186 (70) | 132 (71) | 54 (68) |  |
| AFP, n (%) |  |  |  | 0.579 |
| ~399 | 138 (52) | 96 (51) | 42 (52) |  |
| 400~ | 69 (26) | 46 (25) | 23 (29) |  |
| Unknown | 60 (22) | 45 (24) | 15 (19) |  |
| Age, n (%) |  |  |  | 0.621 |
| ~59 | 129 (48) | 88 (47) | 41 (51) |  |
| 60~ | 138 (52) | 99 (53) | 39 (49) |  |
| Hepatic inflammation, n (%) |  |  |  | 0.797 |
| Mild/severe | 86 (32) | 59 (32) | 27 (34) |  |
| None | 93 (35) | 64 (34) | 29 (36) |  |
| Unknown | 88 (33) | 64 (34) | 24 (30) |  |
| Vascular invasion, n (%) |  |  |  | 0.512 |
| Micro/macro | 76 (28) | 56 (30) | 20 (25) |  |
| None | 153 (57) | 107 (57) | 46 (57) |  |
| Unknown | 38 (14) | 24 (13) | 14 (18) |  |
| Residual tumor, n (%) |  |  |  | 0.918 |
| R0 | 246 (92) | 173 (93) | 73 (91) |  |
| R1/R2/RX | 21 (8) | 14 (7) | 7 (9) |  |

Abbreviations: EZH2, enhancer of zeste 2 polycomb repressive complex 2 subunit; AFP, alpha fetoprotein

^*^*P* > 0.05 indicates that the training set is close to the baseline condition of the validation set and is comparable between the groups.
